# Late Eocene to early Oligocene quantitative paleotemperature record: Evidence from continental halite fluid inclusions

**DOI:** 10.1038/srep05776

**Published:** 2014-07-22

**Authors:** Yan-jun Zhao, Hua Zhang, Cheng-lin Liu, Bao-kun Liu, Li-chun Ma, Li-cheng Wang

**Affiliations:** 1 Ministry of Land and Resource (MLR) Key Laboratory of Metallogeny and Mineral Assessment, Institute of Mineral Resources, Chinese Academy Of Geological Sciences (CAGS), Beijing, 100037, China; 2 China University of Geosciences, Beijing, 100083, China

## Abstract

Climate changes within Cenozoic extreme climate events such as the Paleocene–Eocene Thermal Maximum and the First Oligocene Glacial provide good opportunities to estimate the global climate trends in our present and future life. However, quantitative paleotemperatures data for Cenozoic climatic reconstruction are still lacking, hindering a better understanding of the past and future climate conditions. In this contribution, quantitative paleotemperatures were determined by fluid inclusion homogenization temperature (T*h*) data from continental halite of the first member of the Shahejie Formation (SF1; probably late Eocene to early Oligocene) in Bohai Bay Basin, North China. The primary textures of the SF1 halite typified by cumulate and chevron halite suggest halite deposited in a shallow saline water and halite Th can serve as an temperature proxy. In total, one-hundred-twenty-one T*h* data from primary and single-phase aqueous fluid inclusions with different depths were acquired by the cooling nucleation method. The results show that all T*h* range from 17.7°C to 50.7°C,with the maximum homogenization temperatures (Th_MAX_) of 50.5°C at the depth of 3028.04 m and 50.7°C at 3188.61 m, respectively. Both the T*h*_MAX_ presented here are significantly higher than the highest temperature recorded in this region since 1954and agree with global temperature models for the year 2100 predicted by the Intergovernmental Panel on Climate Change.

Global warming and cooling is the most important environmental problem currently faced by humans. The trigger mechanisms and evolutionary processes of climate change always have been a focus for geoscientists. The Mesozoic and Cenozoic eras are considered to be one of the most important turning points of climate change when Earth's systems transitioned from the middle Cretaceous–early Paleogene typical “green house” conditions to “ice house” conditions after the middle–late Paleogene^1^. Several extreme climate events, such as the Paleocene–Eocene Thermal Maximum (PETM, 55 Ma)^2^, the First Oligocene Glacial (Oi-1; the Eocene–Oligocene, 33.5 Ma)^3^ and the First Miocene Glacial (Mi-1; Oligocene–Miocene, about 23 Ma), have been discovered during this interval. The proposed causes of those events include CH_4_ gas eruption^4^,^5^, volcanic activities^6^, changes in ocean current patterns^7^, and astronomical factors^8^. The drastic climate changes during this interval may be the best analogs for predicting future climate change on Earth^9^,^10^,^11^, however, quantitative paleotemperature data are still lacking, posing an obstacle to further understanding of those events.

The continental and oceanic configuration during the middle–late Eocene was very similar to modern conditions^12^, therefore climatic changes during this interval can provide an analogue for predicting the evolution of future climate. Fortunately, the Bohai Bay Basin, North China has not experienced significant change in latitude for the past 65 Ma^13^,^14^, which offers us an good opportunity to peer into past and future climate conditions.

The Bohai Bay Basin is one of the most important oil and gas basins in China and hosted thick evaporite depositions during the Paleogene. Previous paleotemperature studies in this region mainly focused on palynology and indicated climate conditions changed from dry-hot to warm-humid from the fourth to first members of the Shahejie Formation (Eocene to Oligocene) as suggested by Li et al.^15^ and Xi et al.^16^. Detailed research on the first member of the Shahejie Formation (SF1) indicated a climate shift from cool to warm to cool again^17^, and this change of climate is closely related to the formation of oil and gas source rock in the Bohai Bay Basin^17^,^18^.

The T*h* of fluid inclusions in halite is closely related to the water or air temperature during deposition. This paper quantitatively determines paleoclimate characteristics by analyzing the T*h* of primary, single-phase aqueous fluid inclusions from halite in SF1 (probably late Eocene to early Oligocene). We used the cooling nucleation method to directly measure deposition temperatures of halite. This method has been proven to be very effective and has been widely used in reconstructing ancient climate from different geologic time periods, including Precambrian^19^, Permian^20^,^21^, Eocene^11^, Quaternary^22^, and modern^23^. In addition, a series of experiments^21^,^22^,^24^,^25^ has demonstrated that the maximum homogenization temperature (T*h*_MAX_) of halite fluid inclusions can represent the highest temperature of the water body in which the halite deposited and faithfully reflect the ancient climate. Thus, we chose T*h*_MAX_ from all samples in SF1 as a record of paleotemperatures in the Shulu Sag during this time.

The Shulu Sag is located on the southern edge of the Jizhong Depression of the Bohai Bay Basin (North China), and surrounded by the Hengshui and Xinhe Faults on its northern and eastern margins, and the Ningjin and Xiaoniuchun uplifts along its western and southern margins (Fig. 1). It is a typical half graben-like fault basin^26^,^27^ formed during the Eocene. Halite samples were collected from drill core in the southern Shulu Sag (Fig. 1) and preserved well since there were few volcanic thermal fluid events during the Paleogene to Neogene^28^.

The Shahejie Formation can be divided into four members (SF1, SF2, SF3, SF4) from bottom to top, based on petrologic characteristics (Table 1). The SF1 is late Eocene to early Oligocene in age and consistent with the First Oligocene Glacial (Oi-1) in time. During the deposition of SF1, the Shulu Sag was separated by two uplifts into three sub-sags from north to south. The sub-sag where the study borehole located was a typical continental saline lake with high salinity^29^.

The SF1 discussed in this study is characterized by evaporite sequences consisting of thin clastic layers shaped like bamboo kont (dark-gray mudstone or shale) and argillaceous limestone (Fig. 2). Halite in SF1 occurs in beds within which primary sedimentary structures or textures are well preserved (Figs. 3A and 3B). Chevron or cumulate crystals are widespread (Figs. 3C and 3D) and of which no deformation has been observation. All the evidences suggest that halite samples used in this paper are not altered and their T*h* values can be good climate proxies.

## Results

Primary fluid inclusions in SF 1 halite occur in chevron- or cumulate-type crystals (Fig. 4). Chevron crystals typically form at the bottom of saline lakes with depths less than 60 cm^30^, so the T*h* of fluid inclusions in these crystals is analogous to ancient air temperatures^11^,^21^,^22^,^23^,^25^,^31^. Cumulate crystals usually form at the air–water interface^20^,^21^,^23^ and sink to the bottom under gravity. If cumulate crystals occur in the same beds with chevron crystals, the T*h* of the two types of fluid inclusions can be used to study ancient air temperatures^11^,^19^,^20^,^21^,^22^,^31^. Chevron and cumulate crystals were found in both samples B493 and B1003, which indicates that the halite studied here was deposited in shallow water and the T*h* of fluid inclusions can be used to interpret paleoenvironmental conditions.

In total, about 360 pieces of halite from samples B493 and B1003 have been observed in detail and only 38 pieces of halite are available to T*h* analysis due to primary fluid inclusion in most of the halite pieces are few or absent. The Size of primary fluid inclusions (both of cumulate and chevron crystals) range from 2 to 20 μm and up to 40 to 50 μm. Primary fluid inclusions coexist in both single liquid and gas-liquid phases, and only the single-phase liquid fluid inclusions are chose for T*h* analysis. The T*h* of halite fluid inclusion was tested with the cooling nucleation method, and the results are shown in Table 2.

Sample B493: Sixty-one Th data of primary and single-phase aqueous fluid inclusions were obtained (Fig. 5A). Th values of sample B493 range from 24.4°C to50.5°C and with the average homogenization temperature (T*h*_AVG_) of 39.8°C. Thirteen T*h* values were obtained from fluid inclusions in chevron crystals with T*h*_AVG_ of 39.9°C, T*h*_MIN_ of 24.4°C, and T*h*_MAX_ of 50.5°C. Forty-eight T*h* values were obtained from fluid inclusions in cumulate crystals, with T*h*_AVG_ of 39.7°C, T*h*_MIN_ of 29.5°C, and T*h*_MAX_ of 49.6°C. Sample B1003: Sixty-one T*h* values were obtained (Fig. 5B). Th values of this sample range from 17.7°C to 50.7°C, with the T*h*_AVG_ of 37.3°C Seven T*h* values were obtained from fluid inclusions in chevron crystals, with T*h*_AVG_ of 41.7°C, T*h*_MIN_ of 38.8°C and T*h*_MAX_ of 42.8°C. Fifty-four T*h* values were obtained from fluid inclusions of cumulate crystals, with T*h*_AVG_ of 37.0°C, T*h*_MIN_ of 17.7°C and T*h*_MAX_ of 50.7°C.

## Discussion

Changes of T*h* values from the same sample or even the same inclusion band may be caused by seasonal or diurnal temperature fluctuations^20^,^23^. Consensus is that the T*h*_MAX_ of single liquid phase inclusions can represent the highest brine temperature both at the bottom and at the air–water interface^11^,^22^,^25^.

The ranges of T*h* are different from chevron and cumulate crystals from sample B1003 because the number of fluid inclusions in chevron crystals is smaller than that in cumulate crystals. However, the range and average values of T*h* from these two types of halite crystals in sample B493 are very similar. The T*h* from both samples characterized by normal distribution, and co-occurrence of chevrons and cumulates in the same beds suggests the T*h* is valid and representative. The T*h* values indicate that the water temperature of ancient salt lakes ranged from 17.7 to 50.7°C, and ancient air temperatures had corresponding changes in this region.

As mentioned earlier, the T*h*_MAX_ of single-phase, aqueous fluid inclusions represents the highest air temperature during halite deposition. The T*h*_MAX_ in Shulu Sag during formation of SF1 is 50.7°C, which is 9.2°C higher than the highest temperature recorded since 1954 in this region (local temperature data, and Wu et al.^32^). Given that the T*h*_MAX_ of single-phase aqueous fluid inclusions in halite from modern salt lakes is slightly (less than 5°C) higher than the air temperature^33^,^34^, we infer that paleotemperatures during deposition of the SF1 were at least 4.2°C higher than the temperatures of the past 60 years. Because the study area has not significantly displaced over the last 65 Ma^13^,^14^ and the T*h*_MAX_ values of these two samples (sampling interval is about 160 m) are very similar, we speculate that the higher temperatures state evidenced by Th of halite fluid inclusion in the Shulu Sag may last a considerable time. Combining with the Eocene paleotemperature data from other places in China^11^, it implies that higher temperatures were widespread in eastern China during this interval. This phenomenon of higher temperature is well correlated with the occurrence of large-scale evaporite deposits in the same area during that time. In addition, the average temperature in China has risen by 0.5–0.8°C over the past 100 years^35^, while global temperatures are predicted to rise 1.4–5.8°C by 2100^11^,^36^. This study of homogenization temperatures of fluid inclusions from mid and late Eocene halite in Hubei, China, also indicates that paleotemperatures at the time were 4.6°C higher than today's temperatures^11^. Therefore, it can be predicted that climate warming will continue and not be reversed in the short term.

## Methods

**Figure 2:**
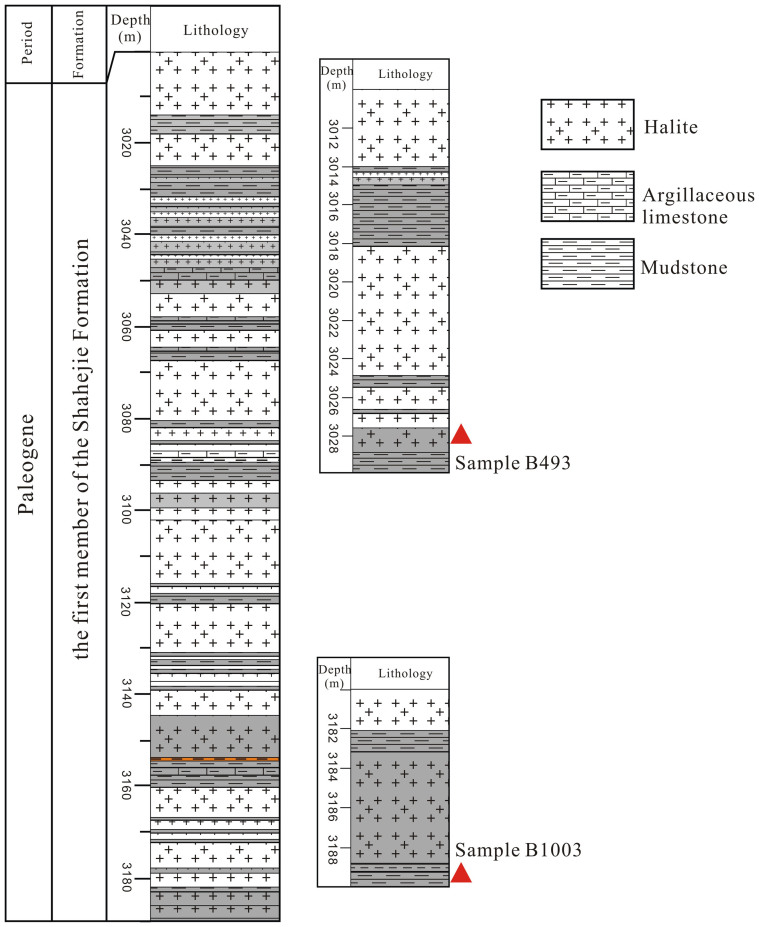
Lithology of the first member of Shahejie Formation (SF1), Shulu Sag, Bohai Bay Basin.

**Figure 4:**
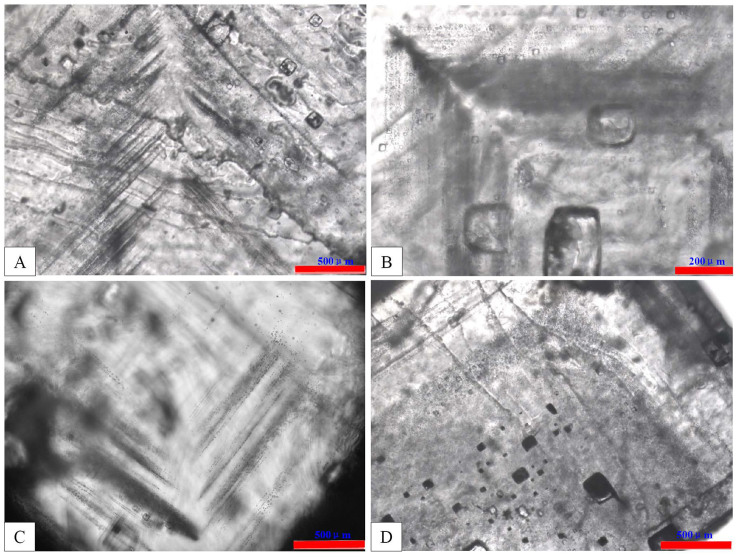
Primary fluid inclusions in SF 1 halite in the Shulu Sag. (A)-photomicrograph of primary fluid inclusions in chevron halite of sample B493; (B)-Photomicrograph of primary fluid inclusions in cumulate halite of sample B493; (C)-photomicrograph of primary fluid inclusions in chevronhaliteof sample B1003; (D)-Photomicrograph of primary fluid inclusions in cumulate halite of sample B1003.

**Figure 5:**
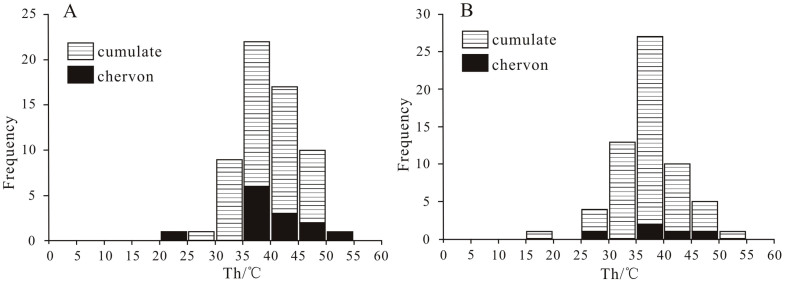
Histogram of homogenization temperature plotted against number of fluid inclusions. (A) -sample B493, 61 Th data of primary fluid inclusions; (B)-sample B1003, 61 Th data of primary fluid inclusions.

**Figure 6:**
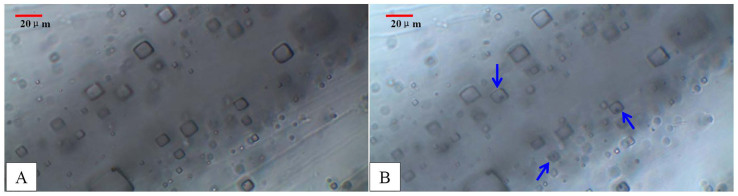
Change of fluid inclusions during the ‘cooling nucleation'process. (A)-primary fluid inclusion without vapor bubble at room temperature; (B)-Vapor bubble formed withinprimary fluid inclusionsafter cooling.

Halite samples were collected from two depths (Fig. 2; 3028.04 m, sample number B493 and 3188.61 m, sample number B1003). Before determining the T*h* of fluid inclusions, halite samples were chosen by XRD (D/max-rA12kw, Rigaku Corporation, Japan). Temperature information is susceptible to distortion due to alteration of halite fluid inclusions by dissolution or heating during sawing. We referred to the methods outlined in Roberts and Spencer^23^, Lowenstein et al.^22^, Benison and Goldstein^20^ and split the halite samples with a hammer and chisel along cleavage planes, into fragments with thicknesses of 0.5 to 1 mm. All the halite fragments were so smooth that changes to the fluid inclusions could be monitored under a microscope during heating and cooling. We observed and photographed these cleavage flakes under the microscope, and documented the occurrence and morphology of primary fluid inclusions. Halite samples were then sealed in self-sealing plastic bags and put into an airtight plastic box. Desiccant was added to the box for moisture protection, and the box was then transferred into a Haier freezer for about 1 week (multiple measurements showed that the temperature was stable at −18°C). After the single-phase fluid inclusions were frozen to nucleate bubbles (Fig. 6), we measured T*h* using a Linkam THMSG600 heating and cooling stage. The heating rate of the stage was first set at 0.5°C/min, but was lowered to 0.1–0.2°C/min when approaching 20°C.

## Author Contributions

Y.J.Z., H.Z., C.L.L. and L.C.M. designed the research in the manuscript. B.K.L. and L.C.W. have been an active participant in the sample collection and processing. Y.J.Z. and H.Z. wrote the manuscript and prepared all figures. All authors reviewed the manuscript.

## Figures and Tables

**Figure 1:**
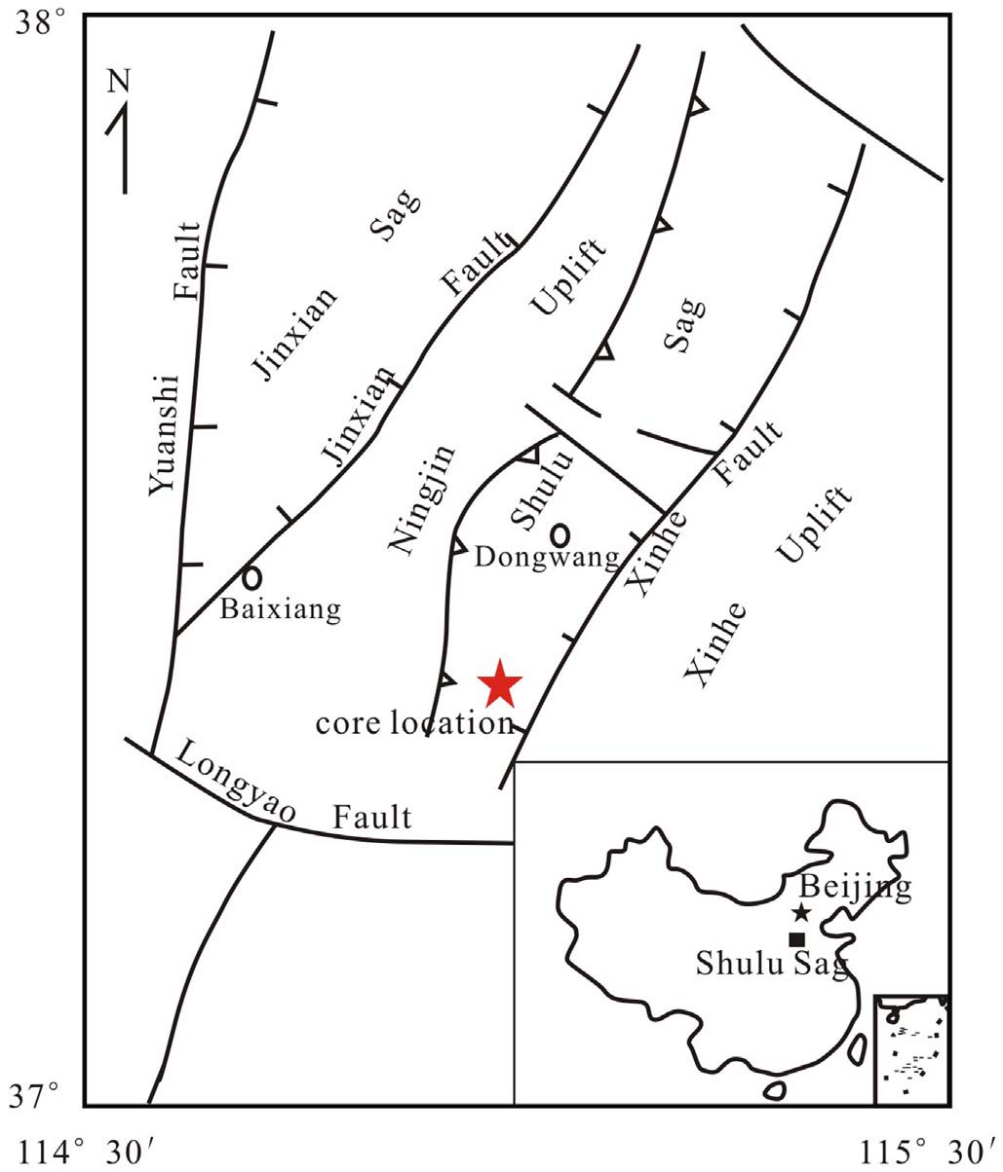
Simplified structural map and location of the study area, the figure was producedusing a base map of ref. 26 and 27.

**Figure 3:**
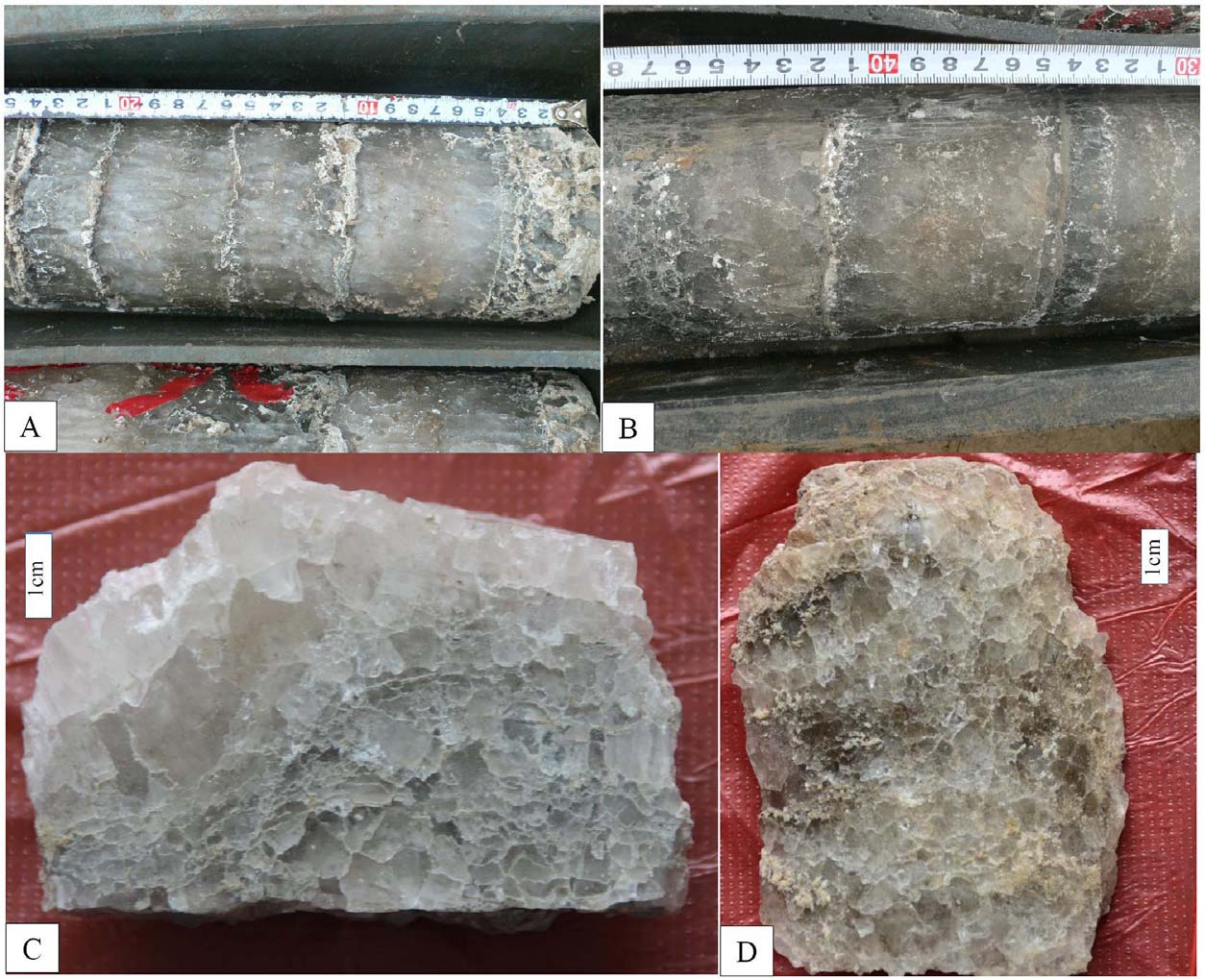
Macrofeature of SF 1 in Shulu Sag, Bohai Bay Basin. (A) and (B), The primary rhythmical textures in evaporite sequence are characterized by the alternating thin clastic beds shaped like ‘bamboo knot' and thick halite bed. (C) (sample B493, 3028.04 m) and (D) (B1003, 3188.61 m), Halite occur in particles and are often overgrown by halite cements.

**Table 1:** stratigraphic characteristics of the Shahejie Formation in Shulu Sag, Bohai Bay Basin, North China

Geological time	Formation	Thickness (m)	Lithology
Oligocene	SF 1	0 ~ 800	The lower part: red and black mudstone, carbonate, gypsum, argillaceous gypsum and salt rock, From north to south in the southern part of the depression, the lithology was marked by limestone - dolomite - gypsum – halite
			The upper part: light grey fine sandstone, siltstone and amaranth mudstone
Eocene	SF 2	0 ~ 400	interbedded brown, purple mudstone and light grey fine sandstone
	SF 3	0 ~ 2200	brown and grey breccia, breccia composed mainly of limestone or dolomite, brownish gray, grey mudstone, local shale and grayish fine sandstone in the upper
	SF 4	500 ~ 1000	Clastic sediments mainly developed around the periphery of the basin, and mudstone sedimentary rocks and mud paste emerged in the center of the depression

**Table 2:** Th statistics of fluid inclusions in SF 1halite

Sample number	Types of crystals	Data of Th(°C)	Th_MAX_(°C)	Th_MIN_(°C)	Th_AVG_(°C)	Th_MEDIAN_(°C)
B493	chevron	24.4;35.2;35.4;35.6;36.3; 37.3;38.3;41.5;43.7;44.5; 47.9;48.0;50.5	50.5	24.4	39.9	38.3
	cumulate	29.5;31.7;33.3;33.4;33.5; 33.7;33.8;34.5;34.7;34.9; 36.5;36.7;36.8;36.9;37.2; 37.2;37.2;37.8;38.2;38.3; 38.5;38.5;38.8;38.9;39.7; 39.7;40.5;40.6;40.6;40.9; 41.0;41.1;41.6;41.7;41.8; 42.0;42.4;43.5;43.9;44.9; 45.7;46.2;47.6;47.6;47.7; 47.8;48.0;49.6	49.6	29.5	39.7	39.3
B1003	chevron	38.8;8.9;49.1;48.1;46.3; 37.6;42.8	42.8	38.8	41.7	48.1
	cumulate	17.7;28.7;29.1;30.0;30.1; 30.2;30.2;32.5;33.0;33.3; 33.5;34.3;34.5;34.6;34.6; 34.8;34.9;35.2;35.2;35.2; 35.6;36.0;36.0;36.2;36.6; 36.8;37.2;37.2;37.2;37.3; 37.5;37.5;37.5;37.7;38.3; 38.5;38.5;38.6;38.8;39.0; 39.3;39.8;40.6;40.6;41.2; 41.3;41.9;43.6;43.7;44.5; 44.6;46.3;48.3;50.7	50.7	17.7	37.0	37.2
